# Effects of a community-level intervention on maternal health care utilization in a resource-poor setting of Northern Ghana

**DOI:** 10.1186/s12889-023-16376-2

**Published:** 2023-08-04

**Authors:** Alex Bapula Kassim, Sam Kofi Newton, William Dormechele, Beatrice Baah Rahinatu, Clement Tiimim Yanbom, Isaac Kofi Yankson, Easmon Otupiri

**Affiliations:** 1 Ghana Health Service, Upper West Region, Wa, Ghana; 2 School of Public Health, Kwame Nkrumah University of Science and Technology, Kumasi, Ghana; 3 Navrongo Health Research Centre, Upper East Region, Navrongo, Ghana; 4 Tumu Midwifery Training College, Tumu, Ghana; 5 CSIR-Building and Road Research Institute, UPO Box 40, Kumasi, Ghana

**Keywords:** Maternal health care, Community-based intervention, Birth preparedness, Ghana

## Abstract

**Background:**

This study aimed to assess the effects of health education and community-level participatory interventions at the community level and the use of community maternal health promoters on the utilization of maternal health care services in poor rural settings of northern Ghana.

**Methods:**

A randomized controlled survey design was conducted from June 2019 to July 2020 in two rural districts of northern Ghana. A multistage cluster sampling technique was used to select the participants. Data were collected from a repeated cross-sectional household survey. Descriptive analysis, bivariate and covariates adjusted simple logistic regression analyses were performed using STATA version 16 statistical software.

**Results:**

At post-intervention, the two groups differed significantly in terms of ANC (p = 0.001), skilled delivery (SD) (p = 0.003), and PNC (p < 0.0001). Women who received health education on obstetric danger signs had improved knowledge by 50% at the end of the study. Women who received the health education intervention (HEI) on practices related to ANC and skilled delivery had increased odds to utilize ANC (AOR = 4.18; 95% CI = 2.48–7.04) and SD (AOR = 3.90; 95% CI = 1.83–8.29) services. Institutional delivery and PNC attendance for at least four times significantly increased from 88.5 to 97.5% (p < 0.0001), and 77.3–96.7% (p < 0.0001) respectively at postintervention. Women who had received the HEI were significantly more likely to have good knowledge about obstetric danger signs (AOR = 10.17; 95% CI = 6.59–15.69), and BPCR (AOR = 2.10; 95% CI = 1.36–3.24). Women who had obtained tertiary education were significantly more likely to make at least four visits to ANC (AOR = 2.38; 95% CI = 0.09–1.67).

**Conclusions:**

This study suggests that the use of health education and participatory sessions led by community-based facilitators could be a potentially effective intervention to improve the knowledge of women about obstetric danger signs and encourage the uptake of maternity care services in resource-poor settings of Ghana.

**Supplementary Information:**

The online version contains supplementary material available at 10.1186/s12889-023-16376-2.

## Introduction

Maternal mortality statistics in developing nations, particularly in Sub-Saharan Africa (SSA), are alarming. The Maternal Mortality Ratio (MMR) in SSA is estimated to be 545/100,000 live births [1]. The inadequacy of obstetric services in countries with inadequate resources is one of the main contributors to this alarming trend [2]. Ghana, one of the SSA nations, has made substantial progress in lowering the MMR from 740 per 100,000 live births in the 1990s to 308 per 100,000 live births in 2017 [3, 4]. Despite Ghana’s gains over the last two decades, research suggests that the inadequacy of qualified labor attendants may be responsible for the country’s persistently high MMR levels [5]. A qualified midwife (nurse, community health nurse, physician assistant) who has had midwifery training, or a medical doctor aiding a woman during labor in a health facility is referred to as a skilled birth attendant. Inadequate maternal health services, low use, limited access to prenatal visits, low facility-based delivery, and low postnatal care have all contributed to Ghana’s current MMR [6].

Many variables influence maternal health care utilization, including negative socio-cultural issues [7, 8], shortage of skilled delivery attendants and any trained midwives declining to work in rural areas [9]. Women in rural Ghana are always at risk during childbirth due to the inequitable distribution of such resources [10]. Even tertiary-care facilities in Ghana, including the Okomfo Anokye Teaching Hospital, have experienced shortage of operating rooms for emergency cesarean sections, forcing women to wait in hospital hallways and increasing their risk of having a difficult delivery [11]. Inequality and low levels of female education are both associated with unwanted maternal health care utilization. Women with less education and those from low-income families are less likely to have access to high-quality care due to monetary and sociocultural barriers [12, 13]. Additionally, there are still barriers to the Community-Based Health Planning and Services (CHPS) initiative, which was created to offer prenatal care at home and support the achievement of Millennium Development Goals (MDG) 4 and 5 [14, 15]. According to Atuoye et al. [16] and Gething et al. [17], rural women find it challenging to obtain facility-based births due to lack of good transportation and bad road networks.

The current maternal health statistics show that more work needs to be done to ensure maternity services are available to all women. Access to, and utilization of services must also improve to provide adequate coverage of good quality. However, geographical access (as in distance), to primary-level health facilities (primarily CHPS, maternity homes and health centers) is generally good, as most rural communities are within 5 to 8 (km) of maternal health care [18].

Community initiatives that target the early initiation of antenatal care can be undertaken to prevent difficulties during childbirth [19]. According to a study conducted in Nigeria, [20] there are clear linkages between socioeconomic variables at the household and community levels and maternal health care use in general [21, 22]. Despite this, there has been a minimal examination of this crucial relationship, with a few outliers [21, 22]. Previous research on maternal health care utilization in Sub-Saharan Africa, notably Ghana, has concentrated mostly on individual-level determinants, with little emphasis paid to contextual and community-level factors.

By evaluating the impact of both individual and community level determinants on the utilization of three components of maternal health services (MHS) in a resource-poor setting of northern Ghana, namely, antenatal care (ANC), institutional-based delivery (IBD), and postnatal care (PNC), this paper addresses a critical information gap. This study’s findings will assist programmers in developing effective interventions and strategies to improve maternal health care utilization at the community level in rural Ghana. The findings will also be used to help build evidence-based policies to address the difficulties that Ghanaian women face during pregnancy.

## Methods

### Study design and population

The effects of the intervention on maternal health choices and outcomes among young married women were evaluated using a randomized controlled assessment approach with cross-sectional pre-post surveys in experimental and control areas. The study population consisted of young married pregnant women. The same pregnant women were later followed up at postpartum using a panel approach. A structured questionnaire was used to collect data on contraceptive awareness and use, coupled with communication on sexual and reproductive health problems, and utilization of prenatal care, institutional delivery and postnatal care services.

### Study setting

The study was conducted in the Sissala East and Sissala West Districts of the Upper West Region, Ghana. The districts were initially one district until 2004 when it was split into Sissala East and Sissala West for effective decentralization by the government. They are largely rural settings located in the eastern part of the Upper West Region [22]. Both districts have one hospital each. The Sissala East district has 44 functional CHPS compounds while Sissala West has 21. According to the 2021 population census, the region had a Total Fertility Rate (TFR) of 3.9 births per woman [22]. The Sissala East district has a total population of 80,619 out of which 39,868 are males and 40,751 are females. While Sissala West has a population of 63,828 out of which 31,556 are males and 32,272 are females [22].

### Program design

The project ‘Strengthening the knowledge of women in the community through health education on the importance and utilization of maternal health services (ANC, facility delivery, and PNC)’ was implemented by community-based Maternal Health Agents (COMHAs) and midwives from June 2019-July, 2020. It was to improve the utilization of maternal healthcare services by pregnant women in two subdistricts in Sissala East and another two in the Sissala West Districts. The project reached out to women who have had a pregnancy experience before the present study. The intervention was delivered to women through a multi-pronged intervention which included: sensitizing family members, community mobilization, and capacity building of frontline health functionaries. One component of the intervention had a continuous counseling strategy using pregnancy classes that were held individually and in groups with pregnant women, their husbands, and other family members. Advocacy meetings were also conducted with influential community members such as village chiefs, local government leaders, and religious leaders to foster an enabling environment for improving pregnant women’s access to, and uptake of maternal healthcare services. Another component of the intervention (awareness, promotion, and use of maternal health services through social and behavior change communication), was implemented by community maternal health service promoters to sensitize pregnant mothers and assist mothers to identify risk factors during pregnancy, delivery and postnatal care. Volunteers from the community who were chosen by the community served as the program implementors. The corresponding author (the District Director of Health Services), one physician (a Family Physician), a midwife, and a health information officer led the training. The lead investigator (Corresponding author) and District Health Administration self-funded the activities.

### Sampling method

There were two and three poor-performing subdistricts (in terms of maternal health indicators) in the Sissala East and Sissala West Districts, respectively. Two subdistricts (one from each district) with similar characteristics were randomly selected out of the five sub-districts. The selected subdistrict (Sakai) of the Sissala East District served as the control arm and all functional health facilities and communities within the selected subdistrict were included in the study. The other subdistrict (Jawia) selected from the Sissala West district served as the intervention arm and again all health facilities and communities were included in the study. Women for the study were selected from communities directly served by the health facilities in the selected subdistricts. Since there were no community-specific population records, the number of women recruited for the study was evenly distributed among the communities. Trained female educators and enumerators were engaged in recruiting and interviewing women. In each community, enumerators worked with the Community Health Officer (CHO) and Community-Based Volunteers to identify and list all eligible women for the study. From this list, a random selection process was used to arrive at the sample size required for the study.

### Sample size

Respondents in the baseline and end-line surveys included pregnant and postpartum women in the age group of 15–49 years from both study groups. Using Cochran’s sample size formula and based on a previous study in a similar context [23], it was assumed that 8.5% of pregnant women and 8.5% of postpartum women were knowledgeable about safe motherhood practices. Using a 5% margin of error, 95% CI, and a nonresponse rate of 10%, the sample size for pregnant women was calculated as indicated below. The same approach was used for postpartum women.

$$\varvec{n}=\frac{\varvec{D}{\left({\varvec{Z}}_{\varvec{\alpha }}\right)}^{2}\varvec{P}(1-\varvec{P})}{{\varvec{d}}^{2}}$$

Where;

**n** = desired sample size.

**D** = design effect which is considered as 2.

**Zα** = standard normal deviate set at 1.96 since a confidence level of 95% is considered.

**d** = degree of accuracy set at 0.05.

**P** = proportion of women (target population) with knowledge of safe motherhood set at 8.5% (0.085) based on previous studies in a similar context [23].

Therefore $$n = \frac{{2{{(1.96)}^2}0.085(1 - 0.085)}}{{{{0.05}^2}}}$$.

**n** = 240.

Considering a nonresponse rate of 10% [24], the final sample size n = 528 per two study arms. Therefore, a total of 264 pregnant women were sampled per the intervention group. The same number of pregnant women was sampled per control group. At the endline, 492 out of 528 women were interviewed, which comprised 250 and 242 women from the control and intervention groups, respectively, as a result of loss to follow-up.

### Inclusion and exclusion criteria

Only postpartum women who had been residents for 12 months of the study and had participated in the intervention from 3 to 12 months in the study area were interviewed. Women who resided in the study subdistricts and had received the intervention throughout the period but might have received ANC, skilled delivery, and PNC services in a different health facility or elsewhere throughout their pregnancy were included in the end-line assessment. Moreover, the end-line evaluation included women who had received the intervention right from the first trimester to the third. Women who voluntarily agreed to participate in the study and signed or thumb-printed written consent were included. However, postpartum women who had not benefited from the intervention during the twelve months were excluded from the end-line survey/assessment. Postpartum mothers whose delivery outcome was either abortion, stillbirth, or death and seriously ill postpartum mothers were exempted from the end-line assessment, this is based on the premise that those mothers might be in emotional distress to answer the questionnaire.

### Study instrument

At baseline, a pretested structured interviewer-administered paper questionnaire, designed in the English language, was used to collect data from participating mothers. At the end line, the same structured questionnaire was used in Open Data Kit and uploaded to mobile phones for data collection; a password-protected cloud-based server was used for data storage. Trained female educators and enumerators conducted interviews with questions on demographic characteristics and various health-related behaviours and utilization of these services: antenatal care, facility-based delivery, and PNC.

### Study variables

This section presents a comprehensive list of both dependent and independent variables utilized in the study, along with their corresponding levels of measurement and variable types. The dependent variable, “Health education intervention”, was a nominal categorical variable with two distinct categories, “Exposed” and “Non-exposed”.

The independent variables consist of several categories, including age, education status, occupation status, household size, marital status, type of marriage, ethnicity, husband age, husband education status, husband occupation, radio/TV ownership, listen to radio, radio discussion, and influenced by a radio discussion. Age and household size were measured as a ratio variable on a discrete scale, while the rest were nominal categorical variables.

The study also measured three variables related to maternal health services utilization: knowledge on maternal health services utilization, attitude on maternal health services utilization, and practice on maternal health services utilization. These variables were ratio variables measured on a discrete scale.

### Statistical analysis

Sample characteristics for women visiting ANC and PNC, as well as those who delivered their babies in health care facilities, were examined using descriptive statistics (means and percentages). At the descriptive analysis level; frequencies, percentages, means, maximums, and minimums were calculated. These statistics provided measures and summaries of the sample and enabled a description of the basic features of the data. Pearson’s Chi-square (χ2) test was used to assess the association between the outcome variables (knowledge, attitude, and practice on the three aspects of safe motherhood) and independent variables (age, marital status, ethnicity, educational status, occupation status, radio/TV ownership, listen to radio, radio discussion, and influenced by radio discussion). T-tests were done to determine differences in mean outcomes of the dependent variables (knowledge score and practice) within the control and intervention arms of the study. This indicated any differences in knowledge and practices as a result of the arms of the study. The mean outcomes of the dependent variables were also compared before and after the intervention and also between controls and interventions. A statistical significance level of p < 0.05 was considered. The dichotomous nature of the outcome variables led to the use of binary logit models. The values ‘1’ and ‘0’ were used to denote outcomes such as yes/no for ANC visits of 4 and > 4. For example, the dependent variables ANC, IBD, and PNC each included two groups (four and more than four visits; institution and home delivery; PNC and no PNC). P-value < 0.05 with a 95% confidence level was used as a cutoff point to declare statistical significance and adjusted odds ratios were used to determine the strength of associations.

### Operational definitions

Knowledge of skilled maternal health services was defined such that women who scored above the mean of knowledge assessment questions were categorized as having good knowledge, and if they scored below the mean, they were considered as having poor knowledge [24, 25].

The attitude was measured by using the Likert scale (1 = strongly agree, 2 = agree, 3 = disagree, and 4 = strongly disagree). A positive attitude was scored by women who responded above the mean of the attitude assessment questions and if below the mean they were categorized as having a negative attitude [24]. Practice (antenatal care, skilled delivery, and postnatal care utilization) was measured such that participants who responded above the mean of the practice assessment question were considered as having a good practice, and if below the mean they were considered as having poor practice [24, 25].

### Ethical consideration

The Kwame Nkrumah University of Science and Technology School of Medical Sciences Committee on Human Research Publication and Ethics (CHRPE) approved the study (CHRPE/AP/190/19). Formal administrative permission was obtained from the Upper West Regional Health Directorate and the Sissala East and Sissala West District Health Directorates to carry out the study. The objectives of the study were explicitly explained to the respondents, the right to withdraw from the study at any time was carefully explained to the respondents, and then written informed consent was obtained from the study participants. To ensure confidentiality and anonymity, the coding of respondents was used.

## Results

### Socio-demographic profile of respondents

Overall, 524 women were interviewed at baseline. This comprised 264 pregnant women sampled in the intervention group, and 260 pregnant women sampled in the control group. At the endline, 492 postpartum women were interviewed, which comprised 250 and 242 women from the control and intervention groups. Women surveyed at both baseline and end-line (two-time points) had similar demographic characteristics. At baseline, most of the pregnant women from both the control (81.0%) and intervention (80.8%) got married when they were aged 18–25 years. Similarly, half of the pregnant women from both the control (50.0%) and intervention (50.0%) groups had no formal education at baseline. At postintervention, most of the postpartum women from both the control (83.6%) and intervention (83.1%) had married at age 18–25 years. The majority of the women from both the control (47.2%) and intervention (42.6%) groups had no formal education in post-intervention (Table 1).

**Table 1:** Socio-demographic Characteristics of Women at Baseline and End line

Variables		Baseline				Post-intervention			
		Control		Intervention		Control		Intervention	
		(n = 264)		(n = 260)		(n = 250)		(n = 242)	
		**n**	**%**	**n**	**%**	n	**%**	**n**	**%**
Age	< 18	4	1.5	4	1.5	-	-	-	-
	18–25	130	49.3	127	48.9	126	50.4	116	47.9
	25+	130	49.2	129	49.6	124	49.6	126	52.1
	Mean age	26.9 ± 7.3		27.0 ± 7.2		27.1 ± 6.6		27.5 ± 6.0	
Age at 1st marriage	< 18	5	1.9	5	2.0	-	-	-	-
	18–25	209	80.7	205	80.4	209	83.6	201	83.1
	25+	45	17.4	45	17.6	41	16.4	41	16.9.
	Mean age	22.5 ± 3.6		22.5 ± 3.5		22.5 ± 3.3		21.7 ± 2.9	
Education Status	No formal education	132	50.0	130	50.0	118	47.2	103	42.6
	Primary	83	31.4	79	30.4	85	34.0	88	36.4
	Secondary	41	15.5	42	16.2	42	16.8	38	15.7
	Tertiary	8	3.1	9	3.5	5	2.0	13	5.4
Occupation status	Housewife	58	22.0	56	21.5	60	24.0	68	28.1
	Farmer	181	68.6	178	68.5	168	67.2	146	60.3
	Employed	8	3.0	9	3.5	5	2.0	11	4.6
	Trader	5	1.9	5	1.9	16	6.4	14	5.8
	Other	12	4.6	12	4.6	1	0.4	3	1.2
Mean household size		5.1 ± 2.1		5.1 ± 2.1		5.0 ± 2.0		4.0 ± 1.6	
Marital status	Married	259	98.1	256	98.5	245	98.0	240	99.2
	Not married	5	1.9	4	1.5	5	2.0	2	0.8
Type of marriage	Monogamy	185	71.4	184	72.2	168	68.6	163	68.8
	Polygamy	74	28.6	71	27.8	77	31.4	74	31.2
Ethnicity	Sissala	250	94.7	245	94.2	244	97.6	231	95.5
	Kassem	1	0.4	1	0.4	1	0.4	1	0.4
	Others	13	4.9	14	5.4	5	2.0	10	4.1
Husband age	< 18	-	-	-	-	**-**	**-**	**-**	**-**
	18–25	34	13.1	32	12.5	36	14.7	23	9.7
	25+	225	86.9	223	87.5	209	85.3	214	90.3
Husband education status	No formal education	166	64.1	162	63.3	157	64.1	146	60.8
	Primary	45	17.4	45	17.6	45	18.4	45	18.8
	Secondary	27	10.4	27	10.5	27	11.0	27	11.3
	Tertiary	21	8.1	22	8.6	16	6.5	22	9.2
Husband occupation	Employed	19	7.3	20	7.8	16	6.5	20	8.3
	Trader	10	3.9	10	3.9	10	4.1	8	3.3
	Farmer	225	86.9	220	85.9	216	88.2	206	85.8
	Other	5	1.9	6	2.3	3	1.2	6	2.5
Radio/TV ownership	Yes	216	81.8	214	82.3	206	82.4	204	84.3
	**Total**	**264**	**100**	**260**	**100**	**250**	**100**	**242**	**100**
Listen to radio	Yes	172	79.6	167	78.0	166	80.6	192	94.1
	**Total**	**216**	**100**	**214**	**100**	**206**	**100**	**204**	**100**
Radio discussion	Yes	131	76.2	129	77.3	132	79.5	178	92.7
	**Total**	**172**	**100**	**167**	**100**	**166**	**100**	**192**	**100**
Influenced by a radio discussion	Yes	117	95.9	5	4	124	93.9	173	97.2
	**Total**	**122**	**100**	**122**	**100**	**132**	**100**	**178**	**100**

### Pre-and post-assessment of women’s knowledge of, and practices related to the utilization of antenatal care, skilled delivery, and postnatal care

The bivariate analysis (Pearson Chi-square test) showed that at baseline, the outcomes were different between the two groups (control and intervention), differed significantly in terms of ANC attendance (p < 0.0001), place of delivery (p = 0.001), and PNC attendance (p < 0.0001).

At post-intervention, there were improvements in the intervention group. The outcomes were different between the two groups (control and intervention), and there was a statistically significant difference in terms of ANC attendance (p = 0.001). In the same vein at post-intervention, the two groups (control and intervention), differed significantly in terms of place of delivery (p = 0.003), and PNC attendance (p < 0.0001) (Table 2).

**Table 2:** Utilization of antenatal care, skilled delivery, and postnatal care services in control and intervention arms at baseline and postintervention

Variables	Baseline			Post-intervention		
	Control (N = 264)	Intervention (N = 260)	p-value	Control (N = 250)	Intervention (N = 242)	p-value
	%	%		%	%	
**ANC attendance**			0.000			0.001
Yes	87.5	79.6		89.1	97.1	
**ANC visits**			0.286			0.277
< 4 ANC visits	16.3	19.9		10.0	13.2	
≥ 4 ANC visits	83.7	80.1		90.0	94.8	
**Place of delivery**			0.001			0.003
Institutional	89.0	82.2		93.4	99.2	
Home delivery	11.0	17.8		6.6	0.8	
**PNC attendance**			0.0001			0.001
< 4 visits	21.6	31.1		17.6	3.3	
≥ 4 visits	78.4	68.9		82.4	96.7	

### Percentage of women who knew about obstetric danger signs at baseline and end line in the control and intervention arms

**Fig. 1:**
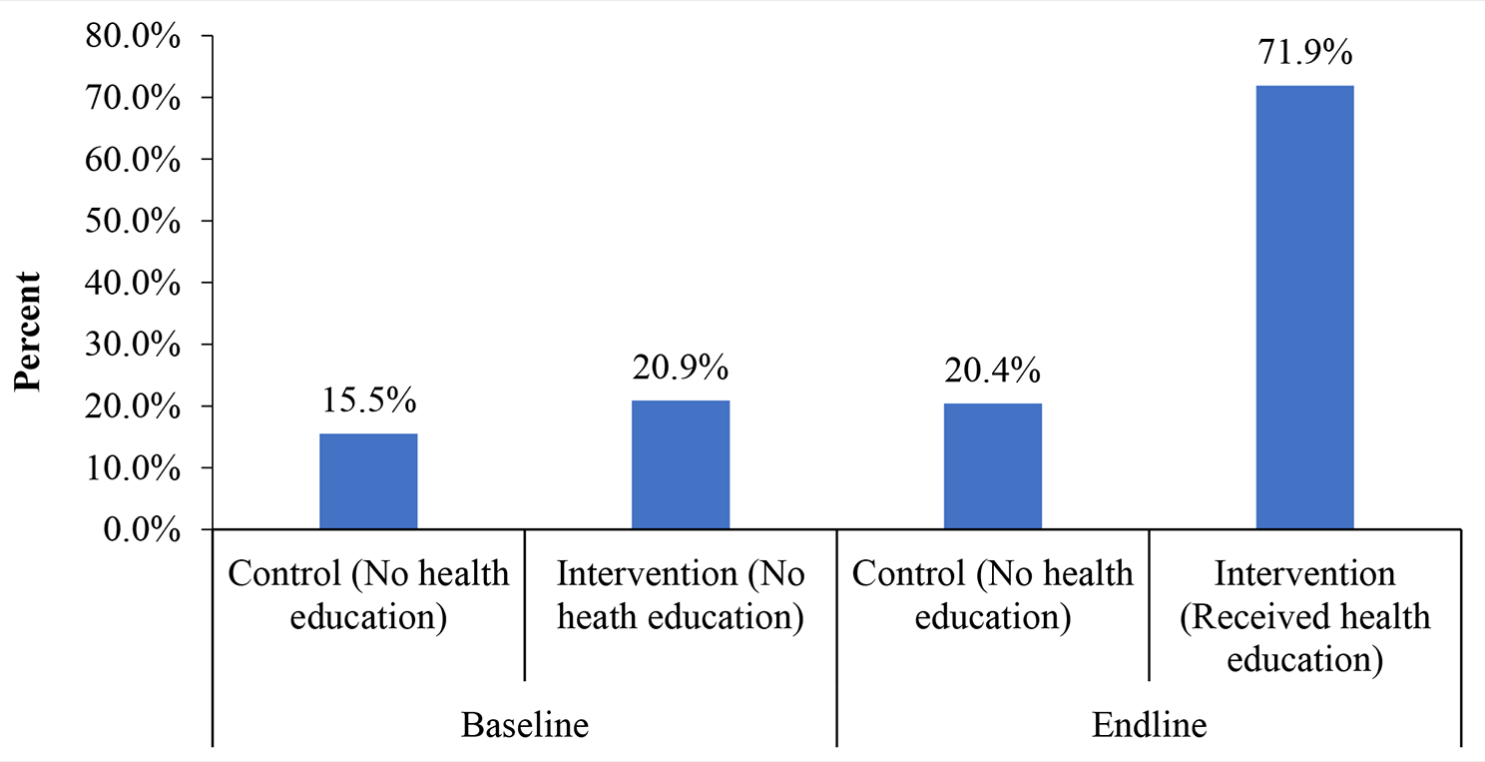
Percentage of women who were knowledgeable on obstetric danger signs, baseline, and end line

The end-line data showed that women who received the health education intervention from Community Maternal Health Agents and midwives about obstetric danger signs had improved knowledge by 50% at the endline compared to women who did not receive the health education (Fig. 1).

### Effect of a health education intervention to improve knowledge of pregnant women on danger signs, complications, and birth preparedness plan readiness

At post-intervention, women who had received the health education intervention (intervention arm) were significantly more likely to have good knowledge on danger signs when compared with women who did not receive a health education intervention (control arm) (AOR = 10.17; [95%CI = 6.59–15.69]; p = 0.0001). Those women who had received the health education intervention (intervention arm) were significantly more likely to be knowledgeable on birth preparedness and complication plan readiness compared to those who did not receive the health education intervention (control arm) (AOR = 2.10; [1.36–3.24); p = 0.001) (Table 3).

**Table 3:** Effect of a health education intervention to improve knowledge of pregnant women on danger signs, complications and birth preparedness plan readiness

Variable	Univariate analysis		Adjusted simple logistic regression analysis	
	OR	(95% CI), p-value	AOR	(95% CI), p-value
Post-intervention				
**Danger signs**				
Control arm (No health education on danger signs)	1 (Reference)		1 (Reference)	
Intervention arm (Health education on danger signs)	10.61	(6.91–16.29); 0.000	10.17	(6.59–15.69); 0.000
**BPCR**				
Control arm (No health education on BPCR)	1 (Reference)		1 (Reference)	
Intervention arm (Health education on BPCR)	2.38	(1.64–3.47); 0.000	2.10	(1.36–3.24); 0.001

OR: Unadjusted Odds Ratio; AOR: Adjusted Odds Ratio; CI: Confidence Interval

### Covariate adjusted simple logistic regression of factors that influenced the utilization of antenatal care, skilled delivery, and postnatal care services

A covariates adjusted simple logistic regression model was used to determine the factors that influenced the utilization of antenatal care, skilled delivery, and postnatal care services after the community-based intervention. The results show that age at first marriage, education status, distance to a health facility, received health education on maternal health services, paid money to staff for ANC and delivery, and application of traditional remedies during pregnancy or delivery were factors that were associated with at least four ANC visit times, skilled delivery and PNC uptake; after adjusting for confounding variables in the model (Table 4).

The odds of at least four ANC visits, skilled delivery, and PNC attendance were significantly higher among women in the intervention group (AOR = 3.49, CI: 1.76–7.83), (AOR = 5.22, CI: 2.88–12.01), and (AOR = 3.82, CI: 1.81–8.97) respectively compared to the control site. Women who had attained tertiary education were 2.38 times more likely to attend ANC at least four times when compared with women with no formal education (AOR = 2.38; [95%CI = 0.09–1.67]; p = 0.022).

Regarding skilled birth attendance, age at first marriage, education status, distance to a health facility, received HIV test results during pregnancy, and application of traditional remedies during pregnancy or delivery were the factors that influenced women to utilize skilled birth services; after adjusting for confounding variables. Women who have attained primary and secondary levels of education were 4.71 and 7.91 times, respectively, more likely to have skilled birth attendance compared to women with no formal education (AOR = 4.71; [95%CI = 1.13–19.54]; p = 0.033) and (AOR = 7.91; [95%CI = 0.86–72.62]; p = 0.031) respectively (Table 4).

Distance to a health facility, receiving HIV test results during pregnancy and paying money to staff for ANC and delivery were the factors that influenced women to utilize PNC services. Women who travelled less than 5 km to a health facility had significantly higher odds of attending ANC at least four times (AOR = 2.58; 95%CI: 1.31–5.07; p = 0.026), skilled delivery (AOR = 3.55; [95%CI = 1.49–8.72]; p = 0.038), and PNC (AOR = 3.47; [95%CI = 1.32–9.01]; p = 0.026) compared to women who travelled more than 5 km to reach a health facility. Similarly, women who received HIV test results during pregnancy were 3.45 times more likely to attend ANC and PNC compared to those who did not receive HIV test results during pregnancy (AOR = 3.45; [95%CI = 1.54 − 7.75]; p = 0.004) (Table 4).

**Table 4:** Covariate adjusted simple logistic regression of factors that influenced the utilization of antenatal care, skilled delivery, and postnatal care services in two selected remote districts of the Upper West Region after the community-based intervention (2019–2020)

Factors	ANC at least 4 times		Skilled birth attendance		Postnatal care	
	AOR (95%CI)	p-value	AOR (95%CI)	p-value	AOR (95%CI)	p-value
**Treatment**						
Control (Reference)	1					
Intervention	3.49 (1.76–7.83)	0.001	5.22 (2.88–12.01)	0.001	3.82 (1.81–8.97)	0.031
**Age at marriage**						
18–25 (Reference)	1		1		1	
25+	0.69 (0.31–1.50)	0.034	3.02 (0.91–10.08)	0.007	1.07 (0.51–2.25)	0.852
**Education status**						
No formal education (Reference)	1		1		1	
Primary	1.16 (0.51–2.66)	0.718	4.71 (1.13–19.54)	0.033	0.66 (0.29–1.50)	0.322
Secondary	1.65 (0.43–6.30)	0.046	7.91 (0.86–72.62)	0.031	0.69 (0.25–1.92)	0.476
Tertiary	2.38 (0.09–1.67)	0.022	1.09 (0.11–10.40)	0.024	0.79 (0.09–6.84)	0.831
**Distance to the health facility**						
Within 5 km	2.58 (1.31–5.07)	0.026	3.55 (1.49–8.72)	0.038	3.47 (1.32–9.01)	0.026
Beyond 5 km (Reference)	1		1		1	
**Received health education on ANC, Skilled Delivery, and PNC**						
No (Reference)	1		1		1	
Yes	5.32 (1.93–14.63)	0.001	0.65 (0.07–5.70)	0.699	0.37 (0.08–1.77)	0.215
**Received HIV test results during Pregnancy**						
No (Reference)			1		1	
Yes	#	#	7.43 (2.00– 27.57)	0.003	3.45 (1.54–7.75)	0.004
**Paid money to staff for ANC and delivery services**						
No (Reference)	1		1		1	
Yes	0.29 (0.13–0.66)	0.003	1.20 (0.37–3.91)	0.761	2.35 (1.10–5.04)	0.028
**Application of traditional remedies during pregnancy or delivery**						
No (Reference)	1		1		1	
Yes	0.37 (0.18–0.76)	0.007	1.60 (0.46–5.61)	0.046	0.57 (0.29–1.14)	0.110

### Effect of a health education intervention on pregnant women’s attitudes towards, and practices related to the use of ANC and skilled delivery services

At post-intervention, women who received the health education intervention on skilled delivery were 3 times more likely to have a positive attitude towards skilled delivery compared to women who did not receive the health education intervention on skilled delivery (AOR = 3.34; 95%CI: 2.12–5.25; p = 0.001). Moreover, at post-intervention, women who received the health education intervention in practice related to ANC and skilled delivery had significantly increased odds to utilize ANC (AOR = 4.18; 95% CI = 2.48–7.04; p = 0.001) and skilled delivery services (AOR = 3.90; 95% CI = 1.83–8.29; p = 0.001) compared to women who did not receive the health education intervention (Table 5).

**Table 5:** Effects of a health education intervention on pregnant women’s attitudes and practice towards the use of ANC and skilled delivery in postintervention

Variable	Univariate analysis		Adjusted simple logistic regression analysis	
	OR	(95% CI), p-value	AOR	(95% CI), p-value
**Post-intervention**				
**Attitude towards ANC**				
Control arm (No health education on ANC)	1 (Reference)		1 (Reference)	
Intervention arm (Health education on ANC)	1.60	(0.94–2.74); 0.084	-	
**Attitude toward Skilled delivery**				
Control arm (No health education on skilled delivery)	1 (Reference)		1 (Reference)	
Intervention arm (Health education on skilled delivery)	4.43	(2.90–6.77); 0.001	3.34	(2.12–5.25); 0.001
**Practices related to ANC**				
Control arm (No health education on ANC)	1 (Reference)		1 (Reference)	
Intervention arm (Health education on ANC)	6.36	(3.90–10.40); 0.001	4.18	(2.48–7.04); 0.001
**Practices related to skilled delivery**				
Control arm (No health education on skilled delivery)	1 (Reference)			
Intervention arm (Health education on skilled delivery)	6.70	(3.33–13.48); 0.000	3.90	(1.83–8.29); 0.000

### Impact of community participation and stakeholder engagement to improve community support to enhance maternal health outcomes

In the intervention area, 83.5% of women made four or more antenatal care visits during their last pregnancy at baseline and its uptake significantly increased to 90.0% (Diff-in-diff=-3.2; t = 29.69; p < 0.0001) at the end line. The Difference-In-Difference (DID) analysis revealed a net improvement of 6.5% in the intervention area with respect to changes in the control area. Regarding the place of delivery in the intervention area, 88.5% of the women had institutional delivery at baseline and its uptake improved to 97.5% at the end line. The analysis revealed a significant net improvement of 9.0% (t = 47.84; p < 0.001) after the intervention in the intervention area with respect to changes in the control area. Moreover, the findings from the end-line survey showed that in the intervention area, 96.7% (Diff-in-diff=-19.40; t = 29.69; p < 0.001) improvement in PNC visits was found while 77.3% improvement among women in the control area. The analysis revealed a significant net improvement of 19.4% in the intervention area over the control area (Table 6).

**Table 6:** Impact of intervention on safe maternal care practices at baseline and post-intervention

Maternal care outcomes	Baseline				Post-intervention			
	Control (N = 264)	Intervention (N = 260)	% Point difference	t (p-value)	Control (N = 250)	Intervention (N = 242)	% Point change	t (p-value)
**ANC Visit**								
≥ 4 ANC visit	83.7	83.5	0.2	18.69 (0.001)	86.8	90.0	3.2	29.69 (0.000)
**Place delivery**								
Institutional delivery	89.0	88.5	0.5	24.89 (0.001)	90.6	97.5	6.9	47.84 (0.000)
**PNC Visit**								
≥ 4 Visit	78.4	77.3	1.1	2.14 (0.115)	82.4	96.7	19.4	2.29 (0.130)

## Discussion

The main issue that this study tried to answer was whether community-level social and behavioral change, interaction with Maternal Health Promoters or Agents and community involvement had an effect on the use of maternal health care. Additionally, if there were any differences between the research arms that were the consequence of other factors. The outcomes of the randomized controlled study showed that the social and behavior modification intervention at the community level significantly increased the use of MHS.

The inferential analysis supported the hypothesis that women who received prenatal health education about the value of using MHS and could remember learning about pregnancy complications were more likely to attend ANC and had a higher likelihood of giving birth in a medical facility than those who did not. The relationship between receiving maternal health care information about pregnancy issues during ANC, having a baby in a hospital, and PNC was also highly significant. This finding is consistent with several other studies on the association between access to health information and the adoption of healthy behaviors. In a similar vein, Mageda and Mmbaga discovered in Tanzania that women who received advice from healthcare professionals to deliver at a healthcare facility had a higher likelihood of doing so [18]. The results corroborate earlier research from Ethiopia [26, 27], Eritrea [28], Zambia, and Sri Lanka [29], which indicated a positive relationship between women’s usage of ANC, institutional delivery, and PNC services and the information they get during maternal health care. Mothers who are exposed to maternal health education/information about pregnancy difficulties have a larger fear, as indicated by Eshete, Legesse, and Ayana, and this can lead them to give birth in a medical institution [30]. This is connected to the notions of perceived severity and cues to action from the Health Belief Model, which claims that people are more inclined to adopt healthy practices when they believe their condition to be severe [31]. In this regard, expectant women who learn about pregnancy problems may become terrified of the abnormalities they might experience if they homebirth.

This is also related to two constructs of the Health Belief Model - perceived severity and cues to action, which state that individuals are more likely to take up positive health behaviors when they perceive a condition to be severe. In this sense, pregnant women who get information on pregnancy complications might be afraid of the complications they are likely to face when they deliver at home.

An improvement in the utilization of maternal healthcare services (at least four ANC visits, institutional delivery, and at least four PNC visits) was noted in the current study. This change is mostly attributable to the effectiveness of the strict intervention measures used. We found a relationship between educational level and propensity to use MHS (ANC, facility delivery, and PNC). Maternal education affects the use of maternal health services like ANC, facility delivery, and PNC, according to numerous research from Bangladesh, [32, 33] as well as other LMICs including Nepal [34], Cambodia [35], Ethiopia [30, 36, 37], Eretria [38], and India. [39].

There are various explanations for the relationship between formal education and MHS uptake. First of all, educated women are better able to understand the health messages that will be distributed during ANC as well as those that are distributed through other media, such as newspapers, billboards, and other forms of mass communication [40]. Second, formal education dispels false notions rooted in many traditions that view some healthy practices as taboos, which might help educated women develop favorable views toward childbirth in a medical institution [40]. Third, a greater level of education is linked to better information processing abilities, increased cognitive skills, and valued sentiments of self-worth and confidence, all of which improve the utilization of health services, according to Fekadu, Ambaw, and Kidanie [36] and Gebregziabher et al. [38]. According to Tsegay et al., formal education is linked to women’s empowerment, which empowers women to make better healthcare decisions and more quickly identifies potential difficulties during pregnancy and delivery [26]. Education improves women’s decision-making abilities and boosts their understanding of how and where to seek the greatest healthcare [41]. Fekadu et al. [36] added that educated women have a better understanding of the risks associated with not giving birth in a medical institution and are more aware of their health. As a result, people are more likely to be informed and select the finest services from the variety of options available to them. To help Ghana reach the Sustainable Development Goals relating to maternal and child health [32], it is essential to invest in female education in rural regions.

Some demographic factors such as mothers’ age and distance to health facilities remain critical and could explain the low uptake of the intervention [42, 43]. Consistent with findings in previous research [42, 44], distance to the nearest health facility remains a challenge for access to and utilization of health services even with community-based approaches. Long distances to health facilities coupled with a lack of transportation in rural areas play a great role in the utilization of delivery services. In most settings of the present study area, people have to carry the laboring mother on a motor tricycle, or on a bicycle to reach a health facility during labor. This highlights the importance of establishing and expanding maternity waiting homes close to where women reside to address long-distance healthcare access issues in the long run. All these perspectives are viewed as crucial in improving the utilization of MNH services. Strategies that address access factors (distance) within community-based interventions need to be considered to enable mothers to reach health facilities.

### Limitations and strengths of the study

The findings from this study provide important considerations for advocacy. There was a likelihood of recall bias since the study required the women to recall previous events. The COVID-19 pandemic has also impacted the research; the study sought to use focus group discussions and pregnancy classes to educate mothers. However, some sessions were canceled due to restrictions that were imposed on the social gathering. This could have also influenced the outcome of the findings. The utilization of a randomized controlled study design with a comparison group and pre-and post-intervention assessment to detect associations is an essential strength of our study. The employment of community maternal health agents in promoting the utilization of maternal health care services is feasible and can be effective, according to the analysis.

## Conclusion

To increase pregnant women’s awareness of and use of maternal healthcare services, the community-based integrated intervention method can be beneficial. In northern Ghana, the maternity care results and women’s household decision-making behavior had a decreased correlation. However, engaging pregnant mothers and family members led to improvements in women’s decision-making and the uptake of maternal health services.

For any community health program to have an impact, it may require an effective social and behavior change, communication drive, and the involvement of community members. Linking community health care workers and Community Maternal Health Agents was pivotal for effective outcomes as demonstrated in this present study. Strengthening community-based services in poor and remote districts in deprived areas through the support of community health workers and Community Maternal Health Agents or volunteers is a potentially effective intervention that contributes to increasing coverage of maternal and newborn health interventions. The observed findings in this study are suggestive of the important role of health promotion campaigns in the improvement of community health systems for remote populations.

The Ghana Health Service in the two districts should use appropriate health education tools to improve knowledge of the importance of the utilization of core maternal health services (ANC, SD, PNC). The implementation of such a strategy should take into account training community volunteers nominated from each subdistrict/community called Maternal health service assistants/Agents (MAHSA). These volunteers should be equipped and linked to health facilities in each area. Moreover, midwives should reorient their caring practices toward more culturally appropriate and evidence-based maternity care. Further research should be conducted to evaluate the effect of increased service utilization rates on staff workload and quality of service in the intervention sites (Preferably through Randomized Control Surveys).

### Electronic supplementary material

Below is the link to the electronic supplementary material.

Supplementary Material 1

## Data Availability

All data generated or analysed during this study are included in this published article as supplementary file 1.

## Abbreviations

ANC: Antenatal Care
AOR: Adjusted Odds Ratio
BPCR: Birth Preparedness and Complication Readiness
CI: Confidence Interval
CHRPE: Committee on Human Research Publication and Ethics
CHO: Community Health Officer
CHPS: Community-Based Health Planning and Services
COMHAs: Community-based Maternal Health Agents
COR: Crude Odds Ratio
CBR: Crude Birth Rate
IBD: Institutional-Based Delivery
MHS: Maternal Health Services
MMR: Maternal Mortality Ratio
MDG: Millennium Development Goals
PNC: Postnatal Care
SD: Skilled Delivery
SSA: Sub-Saharan Africa
TFR: Total Fertility Rate
